# A Study on UVC Photodetector Using Mixed-Cation Perovskite with High Detection Rate as Light-Absorption Layer

**DOI:** 10.3390/nano12071185

**Published:** 2022-04-01

**Authors:** Soo Beom Hong, Hyung Wook Choi

**Affiliations:** Department of Electrical Engineering, Gachon University, 1342 Seongnam Daero, Seongnam-si 13120, Korea; cldkrml@gachon.ac.kr

**Keywords:** high-performance photodetector, mixed-cation perovskite, post-treatment process

## Abstract

In this study, a mixed-cation perovskite ultraviolet (UV) C photodetector was fabricated using a simple formamidinium iodide (FAI) post-treatment process. The fabricated device uses FA_x_MA_1−x_PbI_3_ perovskite as a light-absorption layer and SnO_2_, which has high transmittance in the UVC wavelength region, as an electron-transport layer. The fabricated device exhibited a response of 50.8 mA/W, detectability of 4.47 × 10^13^ Jones, and external quantum efficiency of 53%. Therefore, the approach used in this study is promising for many applications in the UVC wavelength region.

## 1. Introduction

Ultraviolet (UV) rays are detrimental to the human body, causing conditions such as skin redness, aging, and keratitis. UV photodetectors (PDs) have been of long-standing interest [1,2]. In the past, UV PDs have been manufactured using semiconductors with wide bandgaps, such as AlGaN, MgZno, and Ga_2_O_3_. However, this type of UV PD is manufactured using complex and expensive techniques, such as chemical vapor deposition, magnetron sputtering, and epitaxy, hindering their commercialization [3,4,5,6]. Therefore, it is essential to develop a low-cost and relatively simple UV detector. The International Commission on Illumination (CIE) defines three wavelengths in the ultraviolet region: UVA (315–380 nm), UVB (280–315 nm), and UVC (100–280 nm). Currently, most studies are focused on UVA and UVB PDs. Despite being the most dangerous UV light, UVC is studied less [7]. Most of the UVC emitted from the sun is absorbed by the ozone layer before reaching the surface of the earth; thus, a minimal amount of UVC reaches the earth’s surface [8]. However, as the ozone layer is damaged by environmental pollution, the amount of UVC reaching the earth’s surface gradually increases, increasing the effect of UVC on humans, such as blindness and skin erythema [9,10]. UVC is already reaching the earth’s surface from polar regions [11]. UVC is also generated by artifacts such as arc welders, mercury lamps, and lead wires [12]. Therefore, the study of UVC photodetectors is essential.

Organic–inorganic perovskite materials have optical and electrical properties such as low exciton-binding energy due to high dielectric constant, low photocurrent loss due to long carrier-diffusion length, excellent carrier mobility, and high light-absorption ability [13,14,15]. These properties have recently been exploited in sensing applications, including solar-cell applications [16,17]. A UVC PD manufactured using a mixed-cation perovskite prepared by a simple process is expected to exhibit high sensitivity and quick response owing to an improved film surface and stable phase [18]. In addition, it is possible to increase the absorbance by growing crystal grains, which improves the reactivity of the photodetector [19].

An essential factor in the production of UVC photodetectors is the transmittance in the UVC (100–280 nm) region of the electron transport layer. In perovskite-based devices, titanium dioxide (TiO_2_) and [6,6]-phenyl C61 butyric acid methyl ester (PCBM) are often used as electron-transport layers [20]. However, TiO_2_ and PCBM do not transmit light in the UVC wavelength region; thus, a perovskite is not suitable as a light-absorption layer [21,22]. Therefore, using SnO_2_ as an electron-transport layer is essential for fabricating UVC photodetectors [23,24].

This study aims to manufacture a photodetector with higher sensitivity in the UVC region than a conventional photodetector. We used perovskite fabricated using formamidinium iodide (FAI) post-treatment process as a light-absorption layer on a quartz substrate coated with indium tin oxide (ITO). As a result, the absorbance of the prepared FA_x_MA_1−x_PbI_3_ film increased due to the surface improvement of the thin film, phase stability, and crystal-grain growth.

## 2. Materials and Methods

### 2.1. Reagents and Materials

Pb(II) iodide (PbI_2_, 99.999%), sodium dodecylbenzenesulfonate (SDBS), 1-butyl alcohol (99%), ethyl alcohol (≥99.5%), acetonitrile (99.93%), N,N-dimethylformamide (DMF, 99.8%), dimethyl sulfoxide (DMSO, ≥99.9%), 2-propanol (IPA; 75 wt%), 2,2,7,7-tetrakis[N,N-di(4-methoxyphenyl)amino]-9,9-spirobifluorene (spiro-OMeTAD, 99%), bis(trifluoromethane)sulfonimide lithium salt (Li-TSFI; ≥99.0%), chlorobenzene (99.8%), toluene (99.9%), diethyl ethyl (≥99.7%), and 4-tertbutylpyridine (98%) were purchased from Sigma-Aldrich (St. Louis, MO, USA). Formamidinium iodide (FAI), methylammonium iodide (MAI), and methylammonium hydrochloride (MACl) were purchased from GreatCell Solar (Queanbeyan, Australia). A SnO_2_ colloidal solution (15 wt% in water) was purchased from Alfa Aesar (Seoul, Korea). Indium tin oxide (ITO) deposited on a quartz-glass substrate with a thickness of 160 nm was obtained from TMA (Seoul, Korea). All the materials were used without further purification.

### 2.2. Device Preparation

**Figure 1:**
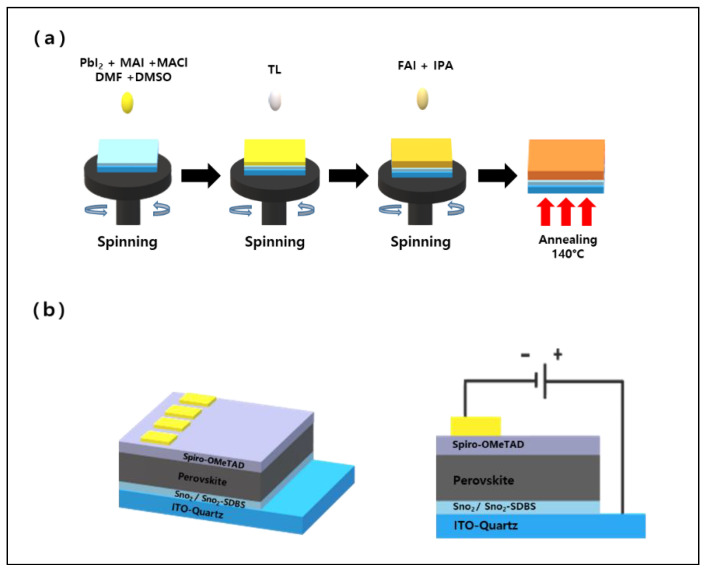
(**a**) Manufacturing process of mixed-cation perovskite FA_x_MA_1__−__x_PbI_3_; (**b**) Schematic diagram of the device architecture on an ITO substrate.

The ITO-coated quartz substrates (8 Ω m^−2^) were sequentially washed with detergent, isopropanol, acetone, deionized water, and ethanol using an ultrasonic bath. The substrate was then dried using a nitrogen gun and treated with UV ozone for 15 min. After diluting 1.2 mL of SnO_2_ colloidal solution (15 wt%) with 5.2 mL of deionized water, the diluted SnO_2_ solution was used to dissolve 1 mg of SDBS to make a SnO_2_-SDBS mixed solution. The SnO_2_-SDBS mixed solution was spin-coated onto the washed ITO at 2000 rpm for 20 s and then annealed at 150 °C for 30 min for use as an electron-transport layer. The substrates were then treated with UV ozone for 15 min prior to the perovskite deposition. The perovskite MAPbI_3_ precursor solution was prepared by mixing PbI_2_ (1.4 mol) and MAI (1.4 mol) in a DMSO:DMF (10:1, *v*/*v*) solvent system. MACl was then added to the prepared precursor solution and stirred for 30 min. The solution was filtered through a 0.45-μm syringe filter just before coating. The post-treatment solution was stirred with IPA and FAI (5, 10, 15, 20, and 25 mg) for 1 h. After cooling at room temperature for 30 min, a spiro-OMeTAD solution [1 mL CB consisting of 72.3 mg spiro-OMeTAD, 28.8 μL 4-tert-butyl pyridine and 17.5 μL Li-TFSI solution (ACN in 1 mL of 520 mg Li-TSFI)] was deposited onto the perovskite film at 3000 rpm for 20 s. Finally, 80 nm of Au was thermally evaporated through the electrode in a high vacuum (2 × 10^6^ Torr). The fabrication method and a schematic of the device are presented in Figure 1a.

### 2.3. Characterization

X-ray diffraction (XRD) was performed on the perovskite films using a Rigaku DMAX 2200 (Tokyo, Japan) with Cu Kα radiation (λ = 1.542 Å) at a scan rate of 5° min^−1^. The surface morphology of the perovskite layer was characterized by scanning electron microscopy (SEM, Hitachi S-4700, Tokyo, Japan). The absorption properties of the perovskite film were evaluated using an Agilent 8453 (Santa Clara, CA, USA) ultraviolet-visible (UV-Vis) spectrophotometer. The electrical properties of the devices were examined using a semiconducting characterization system (2400 Sourcemeter; Keithley, Cleveland, OH, USA) equipped with a probe station (M 150; Cascade, Beaverton, OR, USA). A 254-nm UV lamp (VL6.LC, Vilber, France) was used as the light source for UV irradiation. The mobility and resistivity of the film were measured using a Hall effect-measuring system (HMS-3000, Ecopia, Korea).

## 3. Results and Discussion

### Characteristics of the Prepared Mixed-Cation Perovskite Film

**Figure 2:**
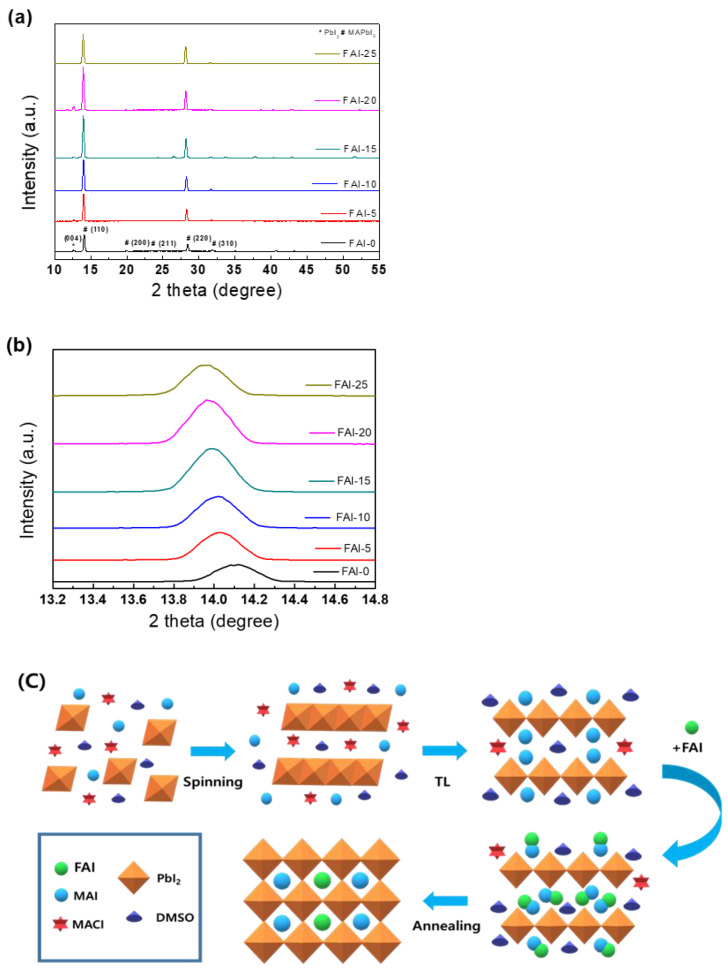
(**a**) XRD pattern of film subjected to post-treatment process with different concentrations of FAI. (**b**) Magnified XRD region for peak (110) of the FA_x_MA_1__−__x_PbI_3_ perovskite film. (**c**) Schematic diagram of perovskite formation prepared via FAI post-treatment process.

The produced perovskite film is defined as FAI-0, FAI-5, FAI-10, FAI-15, FAI-20, FAI-25, depending on the concentration of FAI. Figure 2a shows XRD patterns detailing the effect of FAI treatment in the final formation of the mixed-cation perovskite film. Analyzing the XRD pattern of the film according to the FAI concentration, it can be seen that the crystallinity of the film post-treated with FAI gradually improved. The decreased peak intensity in FAI-25 indicates that an excessive amount of FAI caused grain shrinkage and adversely affected the crystallinity of the film. The highest crystallinity was seen in the FAI-20 sample, and the XRD peak intensity was approximately 2.5 times that of the non-FAI-treated film. Figure 2b shows the XRD peak shift of the (110) plane with increasing FAI concentration. These peak shifts prove that the FA_X_MA_1__−__X_PbI_3_ films were formed.

Figure 2c shows the mechanism of grain-size growth and the formation of FA_x_MA_1__−__x_PbI_3_ with high crystallinity. First, MAI and PbI2 were dissolved in DMSO and DMF and filtered using a 0.45-μm syringe filter. Subsequently, the prepared precursor solution was spin-coated on the ITO substrate on which SnO_2_ was deposited. In the intermediate step, the film was concentrated by evaporation of the solvent, and the spatial steric hindrance of FAI and DMSO prevented the conversion of lamellar PbI_2_ to tetragonal perovskite. Subsequently, through the antisolvent process, perovskite nucleation was accelerated through perovskite-film crystallization and rapid solvent extraction. Finally, FAI was converted to the FA_x_MA_1__−__x_PbI_3_-DMSO phase via ion exchange. Through annealing, a perovskite film with a high crystallinity was formed [20].

**Figure 3:**
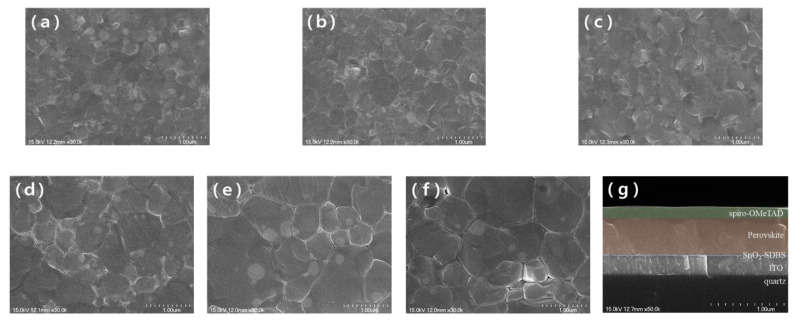
SEM image of the prepared FA_x_MA_1__−__x_PbI_3_ perovskite film (**a**) FAI-0, (**b**) FAI-5, (**c**) FAI-10, (**d**) FAI-15, (**e**) FAI-20, and (**f**) FAI-25, (**g**) Cross section of an optimized device with individual layers highlighted in different colors.

Figure 3 shows the surface morphology and uniformity of the films as observed from SEM. The average particle sizes were calculated as ~220, ~250, ~320, and ~610 nm, for the FAI-0, FAI-5, FAI-10, FAI-15 films, respectively. FAI-20 showed the largest particle size of ~780 nm, and the particle size decreased to ~730 nm for FAI-25. As shown in Figure 3a, the surface of FAI-0 had a very small grain size and nonuniform growth. Compared to the former, FAI-5 and FAI-10 exhibited an increased grain size but lacked uniformity. A significant increase in the grain size and uniformity in the FAI-15 was seen. In FAI-20, the grain size was the largest, and in FAI-25, it can be seen that the grain size was reduced. This increase in the average particle size of the perovskite indicates that the perovskite layer thickened [21]. The grain-size difference was due to the dewetting phenomenon caused by the high concentration of FAI, leading to the shrinkage of the perovskite grains. The shrinkage of these grains also affected their crystallinity, as it created gaps between the grains [22]. This morphological modification of the film demonstrated that the concentration of FAI affected the growth and crystallinity of the film-particle size. Perovskite films with larger particle sizes have been demonstrated to have longer carrier lifetimes and higher absorbances [23,24].

**Figure 4:**
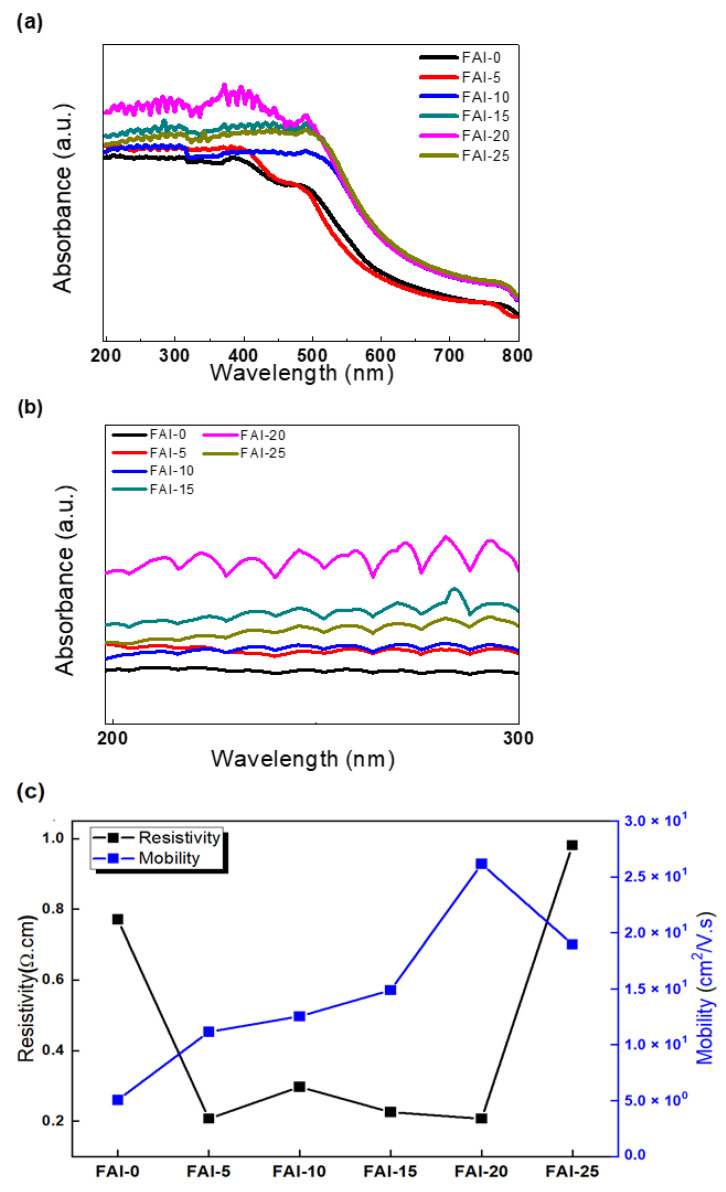
(**a**) UV-Vis absorption spectrum of the prepared FA_x_MA_1__−__x_PbI_3_ perovskite film. (**b**) An enlarged fingerprint region of the UV-Vis absorption spectrum for the UVC region. (**c**) Photoelectric properties of the FA_x_MA_1__−__x_PbI_3_ films.

The UV-Vis absorption spectra of the films post-treated with different concentrations of FAI can be seen in Figure 4a. The spectra were used to investigate the photophysical properties of the films. Compared with the FAI-0 film, the FAI post-treated films exhibited stronger absorbance, with the FAI-20 film showing the strongest extinction coefficient. The decrease in the extinction coefficient of FAI-25 sample was due to grain shrinkage caused by excessive FAI. In Figure 4b, the difference in absorbance according to the FAI concentration in the UVC area was clearly observed by expanding the fingerprint area of the UV-vis absorbance. The films produced by the FAI post-treatment induced the growth of the grains, as confirmed in the XRD and SEM results. Therefore, the extinction coefficient also increased as the size increased, owing to the growth of the crystal grains [25].

Electrical properties including resistance and mobility were measured using a Hall effect-measurement system. The resistivity value of the film not treated with FAI was 0.7707∙Ω cm, and those of the treated films were 0.2074, 0.2975, 0.2258, 0.2072, and 0.9814 Ω∙cm for the FAI-5, FAI-10, FAI-15, FAI-20, and FAI-25 samples, respectively. Mobility was 5.01 cm^2^/V·s for the film not post-treated with FAI and 11.42, 12.56, 14.29, 26.02 and 18.06 cm^2^/V·s for the FAI-5, FAI-10, FAI-15, FAI-20, and FAI-25 films, respectively. As the concentration of FAI increased, mobility increased. In contrast, mobility decreased for the FAI-25. It is interpreted that the addition of FAI increased the diffusion distance and reduced the trap density. The diffusion coefficient can be calculated using the Einstein relation, D = μK_B_T/q, which shows the relationship between the mobility of the current and the diffusion coefficient.

(Boltzmann constant K_B_ = 1.380 6488 × 10^−23^ J/K, the absolute temperature T of the sample, the amount of charge q). The diffusion coefficient is proportional to the diffusion distance. Therefore, high mobility can affect the diffusion distance [26]. Table 1 details the mobility and resistivity values.

**Figure 5:**
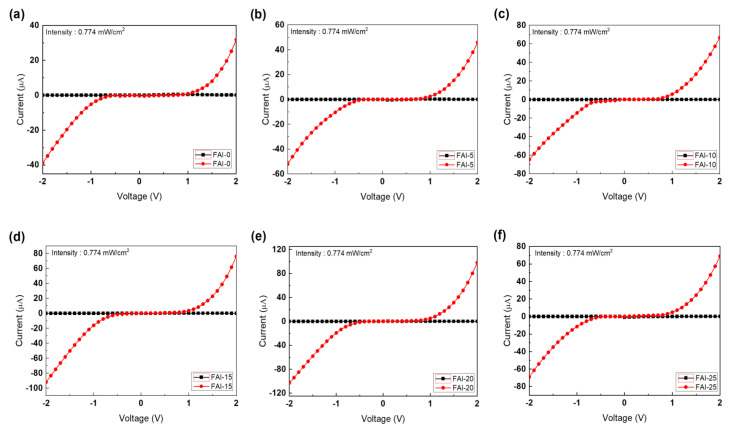
Current–voltage (I–V) characteristics of the FA_x_MA_1__−__x_PbI_3_ perovskite UV PDs post-treated with different FAI concentrations. (**a**) FAI-0, (**b**) FAI-5, (**c**) FAI-10, (**d**) FAI-15, (**e**) FAI-20, and (**f**) FAI-25.

Current–voltage (I-V) curves for PDs fabricated with different concentrations of FAI are shown in Figure 5. All devices were measured in dark conditions and under 254 nm light illumination, with a light intensity of 0.774 mW/cm^−2^. An increased current was detected under 254 nm illumination. This measurement confirmed that a high photocurrent could be photogenerated in FAI-20.

**Figure 6:**
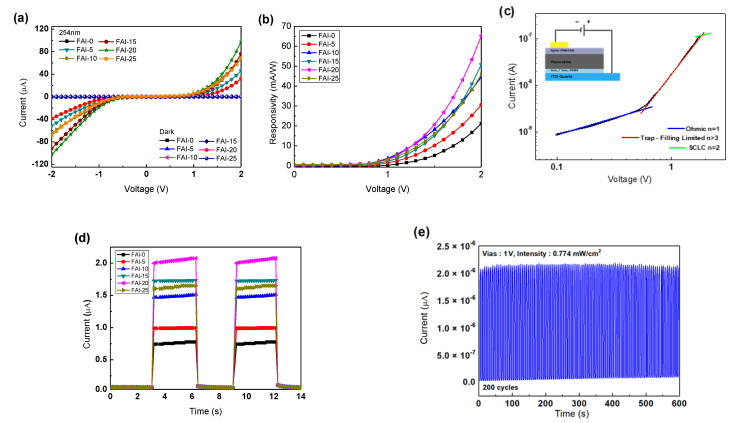
(**a**) Comparison of current–voltage (I–V) characteristics of FA_x_MA_1−x_PbI_3_ perovskite UVC PDs post-treated with different FAI concentrations. (**b**) Responsivity curves of prepared UVC PD. (**c**) The double-logarithmic I–V curve of the device with the highest photosensitivity in dark conditions. (**d**) I-t curve at 254 nm irradiation at −V of UVC PD made with different concentrations of FAI (**e**) Transient light response of the prepared photodetector during 200 ON/OFF switching cycles under illumination of 254 nm light with an intensity of 0.774 mW/cm^2^.

Figure 6a shows the performance of the photodetectors based on different FAI concentrations. The main parameters used to evaluate the performance of UV PD are reactivity (*R*) and specific detectivity (D*) [27]. It is determined by *R* = (*I*_light_ − *I*_dark_)/*AP*_op_, which shows how efficiently the photodetector responds to incident light. (*I*_light_ is the output current under 254 nm UV light, *I*_dark_ is the dark current, A is the active area of the PD, and *P*_op_ is the incident light power intensity). Figure 6b shows the response curve of the photodetector with respect to the FAI concentration. As the bias voltage increased, the *R* value gradually increased. These results can be explained by the increased photon-to-charge conversion efficiency. At a bias voltage of 2 V and 254 nm illumination of 0.774 mW/cm^2^, the *R* values were 21.1, 30.6, 44.5, and 50.8 mA/W, respectively. The highest R value was found from FAI-20, which was 62.3 mA/W. However, in the FAI-25, R was reduced to 45.8 mA/W.

Specific detectivity (D*) is affected by photodetector responsiveness and noise. Therefore, it is defined as $D^{*}=\frac{{\left(A\Delta f\;\right)}^{1/2}\Delta R}{i_{n}}$ (A is the effective area of the PD, Δf is the electrical bandwidth, and i_n_ is the current noise). The three noises that affect D* are the shot noise from dark currents and Johnson and thermal fluctuation “flicker” noises [28,29]. Generally, D* is calculated as $D^{*}=\frac{R}{2qJ_{dark}}$ under the assumption that the shot noise of the dark current has the greatest influence. In this equation, q is the amount of charge, and J_dark_ is the dark current density. This D* can be confirmed by the performance of the signal generated from the light source and the main noise in the dark, and represents the performance of the photodetector. In addition, the *R* and D* values are directly proportional; therefore, in general, the larger the *R* value, the higher the D* value. The D* values of the fabricated devices were 1.84 × 10^12^, 4.54 × 10^12^, 2.46 × 10^13^, 2.97 × 10^13^, 4.47 × 10^13^, and 2.57 × 10^13^ for the FAI-0, FAI-5, FAI-10, FAI-15, FAI-20, and FAI-25 films, respectively. These results demonstrate that the detection power of the samples was superior to that of other perovskite-based PDs [30,31,32].

Another parameter used to evaluate the performance of a sensor is its external quantum efficiency (EQE). EQE is calculated as the number of electrons generated per incident photon (EQE = Rhc/eλ). In the calculation, h is Planck’s constant, c is the speed of light, and λ is the wavelength of the incident light (254 nm). The EQEs at 2 V were 20, 26, 33, 39, 53, and 35%, for the FAI-0, FAI-5, FAI-10, FAI-15, FAI-20, and FAI-25 films, respectively [33].

In Figure 6c, it can be seen that the 𝐼-𝑉 characteristic sharply increases as the voltage bias increases. The 𝐼-𝑉 characteristic trend in the figure is consistent with the space-charge-limiting current in which the trap charge is present [34]. At low bias, the 𝐼-𝑉 current follows an ohmic response. This indicates the formation of an ohmic contact at the interface of the manufactured device. After entering high bias (space-charge-limiting current region), the current is approximately proportional to 𝑉^2+^^𝑛^. where *n* is the improvement factor due to trap filling. In addition, it can be seen that the saturation of the photocurrent occurs due to the Schottky contact formed between the electrodes [35].

Figure 6d shows the time-dependent photoresponse of the PDs measured at a bias voltage of 1 V and a power luminosity of 0.774 mW/cm^2^. When the physical quantity input to the photodetector fluctuates with time, the output of the sensor cannot be changed immediately, and a time delay called the response time is present. The response speed indicates how quickly the output of the sensor can change with input changes. Characterized by rise and fall times, the response rate is defined as the time interval required for the peak value of the output to rise or fall by 10 to 90%. These response speeds and response times are inversely proportional to each other and are the parameters used to evaluate the performance of a photodetector [36]. The rise time (t_rise_) and fall time (t_fall_) of the fabricated devices were calculated as 144 ms/160 ms, 160 ms/167 ms, 220 ms/223 ms, 224 ms/227 ms, 223 ms/272 ms, and 224 ms/261 ms, for the FAI-0, FAI-5, FAI-10, FAI-15, FAI-20, and FAI-25 films, respectively. These results are superior to those of metal-oxide-based photodetectors [37]. Fabricated devices will have consistent results with longer fall times. This is because numerous traps in the active layer briefly capture photocarriers before they are released to contribute to the circuit current, thereby prolonging the fall time.

The operational on/off repeatability of the photodetector using mixed-cation perovskites post-treated with FAI-20 as the light-absorption layer is shown in Figure 6e. The photodetector showed stable performance over 200 ON/OFF repetitions at an interval of 3 s. The 2.08 μA photocurrent in the first iteration was 2.13 μA after 200 iterations. These results demonstrate the excellent photostability of the fabricated photodetectors.

The parameters of the mixed-cation perovskite UVC PD proposed in this study show similar values or better than those of the PDs published in other studies. (Table 2).

## 4. Conclusions

The fabricated mixed-cation perovskite-based photodetector was fabricated through a low-temperature solution process and simple FAI post-treatment. This study shows the improvement of the photosensitivity of perovskite films with the concentration of FAI. It also contributed to the high detectability of devices using mixed-cation perovskite as the light-absorbing layer. The fabricated device exhibited a response of 50.8 mA/W and an EQE of 53%. It also had a detection rate of 4.47 × 10^13^ Jones, superior to other perovskite-based PDs. Consequently, the PDs fabricated in this study provide a promising solution for UVC detection.

## Figures and Tables

**Table 1:** Comparison of resistivity and mobility of FA_x_MA_1__−__x_PbI_3_ films.

	FAI-0	FAI-5	FAI-10	FAI-15	FAI-20	FAI-25
Resistivity (Ω.cm)	0.7707	0.2074	0.2975	0.2258	0.2072	0.9814
Mobility (cm^2^/V.s)	5.01	11.42	12.56	14.29	26.02	18.06-

**Table 2:** Comparison of important parameters of various recently studied UV detectors.

Device Structure	Detectable Light (nm)	Method	Voltage (V)	Responsivity (mA/W)	Detectivity (Jones)	EQE (%)
ITO/SnO_2_/(FA)_x_(MA)_1__−__x_PbI_3_/Spiro-OMeTAD/Au [this study]	254	Solution	2	50.8	4.47 × 10^13^	53
CsPbBr_3_–Cs_4_PbBr_6_/FTO/TiO_2_/MAPbI3/Spiro/Ag [38]	254	Vapor	0	49.4	1.2 × 10^12^	-
Au/MAPbCl_3_/Au [39]	255	Slow evaporation	5	450	-	219
Au/MAPbBr_3_/Au [39]	255	Slow evaporation	5	300	-	146
Au/MAPbI_3_/Au [39]	255	Slow evaporation	5	120	-	58
SRO/BTO/ZnO/Ag [40]	260	Antisolvent vapor-assisted crystallization	3	22.1	1.2 × 10^11^	10.3
Au/CsCu_2_I_3_/Au [41]	265	pulsed laser	2	37.7	8.1 × 10^10^	17.9
Au/Ti/MAPbCl_3_/Pt [42]	365	Inverse temperature crystallization	15	46.9	1.2 × 10^10^	-
Au/FTO/SnO_2_/Cs_2_AgBiCl_6_/TFB/Au [31]	370	Vapor	−5	9.68	1.11 × 10^12^	-

## Data Availability

Not applicable.
